# Did the events following the COVID-19 outbreak influence the incidents of violence against hospital staff?

**DOI:** 10.1186/s13584-021-00471-z

**Published:** 2021-06-17

**Authors:** Fuad Basis, Kobi Moskovitz, Shay Tzafrir

**Affiliations:** 1 Rambam Medical Campus (RMC), the Technion Faculty of Medicine, Haifa, Israel; 2 RMC Administrative Department, Haifa University, Haifa, Israel; 3 School of Business Administration, Haifa University, Haifa, Israel

**Keywords:** Violence, COVID-19, Sympathy, Medical staff, Trust, Communication

## Abstract

**Background:**

During the COVID-19 outbreak, (March 1 - June 15, 2020) citizens expressed sympathy and gratitude towards medical staff through the media, while the entire hospital staff faced the same danger of infection as other citizens. This might have made hospital staff develop sympathy, understanding for the patients` and family’s needs, and a better communication.

**Objectives:**

To investigate if there is a relation between the mutual change in attitude between citizens and hospital staff during the first COVID-19 outbreak, and the incidence of violence cases.

**Materials and methods:**

This is a cross sectional study conducted at Rambam Medical Center (RMC) in Israel. The data about the number of violence cases were collected from the security department, and the data about hospital wards activity were collected from the hospital Business Intelligence (BI) software.

The number of violence cases in relation to the number of Emergency Department (ED) visits, admissions to hospital wards, and length of stay (LOS) were compared during the COVID-19 outbreak to the corresponding period in 2019 using the T- test. The difference in the incidence of violence between general population and people with a psychiatric or social disorder (like drug abuse and criminal background) in both periods were also compared using the Fisher exact test.

**Results:**

During the first COVID-19 outbreak, there were 6 violence cases against medical staff out of 24,740 visits to the ED, vs. 21 cases out of 30,759 visits during the same periods in 2019 (*P* < 0.05). There were 19 violence cases in the whole hospital with 14,482 admissions in 2020 vs. 51 violence cases of 17,599 admissions in 2019 (*P* < 0.05). Violence against security guards in the entire hospital dropped from 20 to 11 cases, and in the set of the ED, from 13 to 4 cases in both periods respectively. A 20 % decrease in the number of visits to the ED, might have influenced the average LOS during the study period, 2020 compared to 2019 (4.4 + 0.45 vs. 5.4 + 0.36 h. (*P* < 0.001). The ratio of violence among general population vs. people with a psychiatric or social background revealed a non-significant change in both periods (*P* = 0.75 and *P* = 0.69) respectively.

**Discussion:**

The COVID-19 outbreak supplied some evidence that a change in environmental conditions, trust, waiting time, personal attitude and communication might have reduced violence against hospital staff.

**Conclusions:**

Except for violence coming from patients with psychiatric or social disorders, most other violence cases might be reduced if the environment conditions and attitudes of both citizens and staff are improved.

## Introduction

Violence against medical staff and other employees in hospitals is a concern in many countries [1, 2]. Currently, many researchers agree that at least three main clusters of factors lead to the development of these violent episodes, which include: environmental factors, patient-related factors, and factors related to communication between medical staff and patients or families [3–5].

Violence against medical staff and security guards in the Emergency Department (ED) comprises a cardinal part of the violence cases in hospitals [6]. Many researchers agree that an inconvenient environment and a crowded ED may cause frustration and stress among medical staff which can be reflected on the patients and their families and can increase the probability of violence [7].

Patient-related factors such as acute illness, fear of the unpredicted and sometimes, extreme stress can cause violence against hospital staff. Respectful communication, including active listening between medical staff and other hospital employees, is important. Also, the interaction of these factors (environment, patient, and communication) can intensify the aggression. These factors may make both the patient and his family members intolerant of any inconvenience or any delay in the work-up waiting time [8]. Expressing empathy, humility, and honesty towards the patient and his family may increase the trust between patient and medical staff and could therefore, increase patients` subjective well-being [9, 10]. However, staff frustrated by the conditions imposed on them by their superiors and the system, would probably have difficulty expressing patience and empathy towards patients and their families [11].

Some unique populations are more violent than the standard population. Patients under alcohol/drug influence, patients with a criminal background (social problems), patients suffering from psychiatric illnesses or personality disorders may be more prone to frustration and have a lower threshold for violence. This could explain why most of the violence cases come from these populations [12].

According to the 676 questionnaires conducted among nurses and physicians by Shafran Tikva et al., the staff stated that 27 % of the cases were due to hospital conditions and waiting times and in 39 % of the cases, the staff behavior contributed to violent episodes [13]. Others found that the unmet demand of the patient and deficient staff number were the leading reasons for aggression [14].

Trust between patients and their families plays an important factor in reducing violence against medical staff. Sharma et al., in their study found that only 4.7 % of the participants attributed violence to financial mistrust, while 45.2 % indicated that increasing the trust between patients and medical staffs through treating the patient with care and politeness is very important in decreasing the rate of violence. Furthermore, 7.6 % of the participants emphasized respect to the doctor and care for his/her job as very important, in reducing violence against medical staff [15].

During the COVID-19 outbreak, both patients and hospitals` staff faced the same danger of being infected. The Israeli Ministry of Health (MOH), and the official media, fed social media with ongoing information about the number of people infected and dead from the disease, which made fear and panic visible to the public and the RMC hospital and other hospitals` staff. This led the RMC management to recruit volunteers to work in the “Corona Department”, and so did many other hospitals in Israel.

This attitude of the hospital staff in many hospitals caused the medical staff, and volunteered staff working with these patients, to earn sympathy, trust and respect from the public through the media. During the COVID-19 outbreak, a positive coverage of the medical teamwork was experienced many times. As a gesture of gratitude, the people applauded the medical staff in the country after a pre-arranged coordination through the social networks and it was broadcast live on television. Furthermore, to express their gratitude to the hospital`s staff, the Israeli air force, using light aircrafts, performed a flight over all the hospitals in the country [16]. Similarly, 120 yachts and ships made a flotilla along the state’s shores for the same purpose. The COVID-19 pandemic was perceived as an enemy, and the medical staff was perceived as soldiers who endanger their lives to protect others, as in wartime [17]. In many countries in Europe also, and in the rest of the world, people expressed their gratitude by applauding health workers, because of risking their lives in treating COVID-19 patients [18].

This study was determined to know whether or not there might be a relation between the particular sympathy attitude that the political leaders, the media, and the people expressed, and the incidence of violence against hospital staff. perhaps the findings of this study might supply some evidence for the requirements needed to prevent violence, if this was indeed the case.

### Objectives

To investigate if there is a relation between the mutual change in attitude between citizens and hospital staff during the first COVID-19 outbreak, and the incidence of violence cases.

## Materials and methods

This cross-sectional study is conducted in the RMC, a tertiary hospital with 1017 beds in the north of Israel. It compares the number of order disruption and violence cases, separately, against hospital staff from March 1 to June 15, 2020 (the first COVID-19 outbreak), to the same period in 2019. The data are separated also as those against medical staff or those against other personnel, mainly security guards. The concentrated data is sent to the RMC, by the Israeli MOH security authority every six months, according to monthly reports from the RMC security department.

Order disruptions are defined as those disorders that do not involve violence against hospital staff. These cases among other things, may include shouting or corruption of property (like when a family member dies), shouting because of long waiting time or even an argument with the parking plot ushers, which is not directed against a specific hospital staff member. Most of these cases end peacefully. Unfortunately, there is no detailed data about the specific kind of order disruptions, while violence against medical staff or against security guard, is divided into three categories: threat, verbal violence or physical violence.

The data detailing the number of visits to the emergency department (ED), the length of stay (LOS) in the ED as well as in other departments and the admissions to the RMC on a daily basis from March 1 until June 15, 2020 (107 days) was collected from the Business Intelligent (BI) hospital computerized software. These data were compared to data of the same period in 2019.

Since the data were collected on a daily basis, it was assumed that the variances have a normal statistical distribution and a T-test was suitable for comparison. Also, the Fisher exact test was used to perform the comparison of the number of order disruptions or violence cases in both periods, as the study dealt with small numbers in both periods of 2020 and 2019.

On applying to the hospital’s IRB, since this study as it is an epidemiologic one, and does not involve information about patients details or their electronic medical records, Helsinki permission is not needed.

## Results

On March 1, 2020 after the detection of the first case of COVID-19, the MOH encouraged citizens` isolation and suggested that citizens should limit their visits to community clinics, except for urgent cases. Families of patients were also instructed to restrict their presence in the hospital to one family member at a time. Citizens complied with these instructions and most of the time stayed out of the hospital departments as well as the ED, and only came to check the progression of the treatment from time to time.

**Fig. 1:**
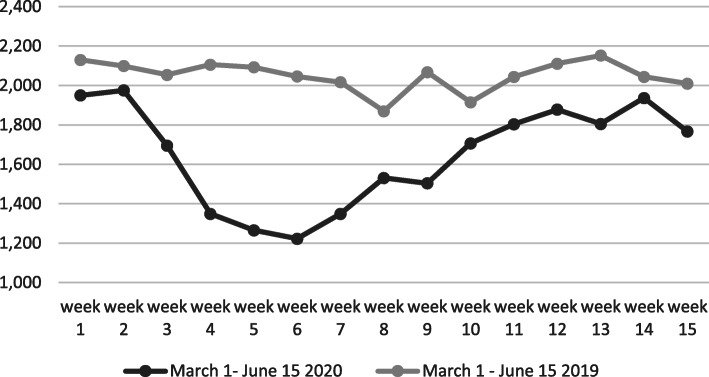
The number of referrals to the ED during the COVID-19outbreak in comparison to the same period in 2019 on weekly basis

After these instructions, from March 1 to June 15, 2020; during 107 days, a decline in the number of visits to the ED was witnessed by 21.7 % in comparison to the same period in 2019 (23,074 vs. 29,487). Using the T-test, the decline of the number of visits to the ED on weekly basis was very significant t (106) = -8.37135, *P* < 0.001 (Table 1; Fig. 1).

**Table 1:** The average number of visits to the ED on a daily basis from March 1 to June 15, 2020 vs. the same period in 2019

Duration	No ‘of visits to ED	Average daily visits	SD	*P*- value
March 1 – June 2020	23,074	213	52	*P* < 0.001
March 1 – June 2019	29,487	273	51	

There was also a decline in the number of admissions to the hospital wards (calculated on a daily basis) by 17.7 % during March 1 to June 15, 2020 in comparison to the same period in 2019. (14,482 vs. 17,599) The results of the T-test showed that this decline was highly significant. t (106) = 5.2858, *P* < 0.001 (Table 2).

**Table 2:** The average daily admissions to the hospital wards from March 1 to June 15, 2020 vs. the same period in 2019

Duration	No. of admissions to hospital	Average daily admissions	SD	*P*- value
March 1 – June 2020	14,482	135	40	*P* < 0.001
March 1 – June 2019	17,599	164	41	

The LOS in the ED on a daily basis was examined and the average daily LOS from March 1 to June 15, 2020 was found to be 5.31 ± 0.75 h. when compared to 6.70 ± 0.77 h. in the same period of 2019.

The result of the T-test showed that the LOS in the ED, during the first COVID-19 outbreak in 2020, was significantly lower than that in the same period in 2019. t (106) = -13.2014, *P* < 0.00.1. The average LOS in the hospital wards on a daily basis was 5.2 ± 1.23 days during the first COVID-19 outbreak in comparison to 5.07 ± 1.06 day in the same period in 2019. The result of the T-test showed that the difference is statistically non-significant t (106) = 1.05653. (*P*-value = 0.145977). This means that the LOS in the medical wards was not influenced by the COVID-19 outbreak despite a reduction in the hospital occupancy, and workload.

From March 1 to June 15, 2020, there were 14,482 admissions to the hospital wards with 84 cases of order disruption compared to 17,599 admissions with 208 order disruptions the same period in 2019. There was a decline of 17.7 % in the number of admissions, and there was a decline of 83.6 % in the number of order disruptions. Comparing the proportions, the results showed that the Fisher exact test statistic value is < 0.00001 and is significant at p < 0.01. In other words, the decline in the number of order disruption is beyond the proportionate reduction in the number of admissions. Unfortunately, we have no detailed data about the part of the ED in these disruptions.

There were 19 cases of violence against medical staff in the hospital during the pandemic period compared to 51 cases in the same period in 2019 (62.7 % reduction). The ratio (proportion) of violence cases against hospital staff was compared in relation to the reduction in the number of admissions in both periods (17.7 %), using the Fisher exact test, it was statistically very significant and beyond the proportionate reduction in the number of admissions. (The Fisher exact test statistic value was < 0.0001. The result is significant at *p* < 0.01) (Table 3).

**Table 3:** Comparison of violence cases against medical staff during the COVID-19 outbreak vs. the same period in 2019, in both hospital and Emergency Department respectively

	Hospital			Emergency Department	
	Admissions	Order disruptions	Violence cases	Visits	Violence cases
March 1 until June 2020	14,482	84	19	23,074	6
March 1 until June 2019	17,599	208	51	29,487	21

There was a decline in 21.7 % in the number of visits to the ED (23,074 vs. 29,487) and a 71.4 % decline in the violence cases (6 vs. 21) against medical staff. The result of the Fisher exact test statistic value is 0.031 and is significant at *p* < 0.05. Again, the proportionate decline in the violence cases is beyond the decline in the number of visits to the ED.

The violence cases against security guards in the entire hospital dropped from 20 cases in March 1 until June 15 2019 to 11 cases in the parallel period during the first COVID-19 outbreak, and that in the ED dropped from 13 to 4 cases only.

According to the statistics of this study, 54 % (10 of 19) of the violence cases in the hospital during the COVID-19 outbreak were due to alcohol/drugs, psychiatric disorder as well as criminal background, when compared to 60 % (31 of 51) in the same period in 2019, the result of the Fisher exact test statistic value of 0.8253 is not significant at *p* < 0.05. For the result obtained for the general people, without psychiatric or social problems, involved in violence in the whole hospital (9 of 19 vs. 20 of 51) the Fisher exact test statistic value is 0.806; and the result is not significant at *p* < 0.05. This suggests that the change in the ratios of both groups with psychiatric or social background and regular population, during both periods in relation to the total number of violence cases was unchanged, or might not be influenced by the change in the situation.

In parallel, during the COVID-19 outbreak, there were 6 violence cases in the ED out of 19 cases in the whole hospital (31 %), in comparison to 21 cases in the ED out of 51 cases (41 %) in the whole hospital in 2019. Although there was a decline in the total number of violence cases in the ED during the COVID-19 period, the change in the ratio was not significant. The Fisher exact test statistic value is 0.7966 and it is not significant at *p* < 0.05. This indicates that the proportion of violence cases in the ED compared to that in the whole hospital was not changed significantly.

The proportionate reduction in the number of violence cases among regular people, using the Fisher exact test, was statistically significant. (The Fisher exact test statistic value is 0.0201. The result was significant at p < 0.05) (Table 3), and it was beyond the decline in the number of visits. There was no case out of the 6 cases that general population (without psychiatric or social disorders) were involved, when compared to 10 out of 21 (47.6 %) in the same period in 2019. Although a Fisher test is hard to perform (zero cases in the COVID-19 outbreak), however, in all cases of violence in the set of the ED, patients with psychiatric or social background were involved.

## Discussion

During the first COVID-19 outbreak, there was a gradual decline in the number of admissions to the hospital wards as well as a decline in the number of visits to the ED. The proportionate reduction in the violence incidents was statistically significant and beyond the reduction in the number of admissions to the hospital and visits to the ED. Despite the significant decline in the number of violence cases during the COVID-19 period, in this current study, the ED remains the main department suffering from violence against medical staff (31 % of all cases; or 6 of 19) in the rest of the departments of the hospital.

Shafran-Tikva et al. in their study in a tertiary hospital found that nurses in the ED were 5.5 times more at risk of being exposed to violence than nurses in the internal medicine departments [19]. It was assumed that this may be partially due to the working conditions in the ED; that is, the exposure of the medical staff to patients with stress or either to patients with psychiatric or social background, that usually are not admitted to the medical wards. Furthermore, the medical staff in the medical wards become familiar with the patients and their families and the communication between them becomes more personal.

There is also a reduction in the number of order disruptions during the COVID-19 outbreak (84 cases), compared to the same period a year before (208). It seems that there is a significant decline in the number of cases, however, it is hard to make statistical conclusions, because the out-patient clinics, during the COVID-19 were almost closed, and the number of visitors was decreased, a parameter that is hard to measure.

The reduction in the workload on the medical staff was thought to cause a significant reduction in the LOS of patients in the ED. Since the LOS in the same period 2019 was 7.4 h., it seems that one-hour reduction in the LOS in the ED is not the main reason for the reduction of violence in the ED.

Furthermore, there was a 21.7 % reduction in the number of visits to the ED, and citizens were considerate and followed the instructions of the guards and the MOH instruction. In fact, in most cases, family members stayed outside the ED and only one person entered the ED at a time and stayed for a short time. This caused a significant change in the physical conditions of the work environment. Furthermore, the reduction in ED workload caused both patients and medical staff to be tolerant of each other with regard to their behavior and needs, and this could have influenced the communication and trust between both parties.

As mentioned previously, similar to the reduction in the amount of violence in the ED, there was a significant reduction of violence in the rest of the hospital wards. Although the LOS of patients did not change in the medical wards, the change in the work environment and the workload might have influenced the behavior of both patients and staffs. Furthermore, in this pandemic, both medical staff and citizens faced the same danger of getting sick. Hence, it was thought that this led medical staff to be more tolerant and compassionate, and vice versa.

This study assumed that the change enforced on both citizens and medical staffs influenced the triggers for violence mentioned in previous literature, for the better [20]. A change in the physical conditions [21], the workload and the waiting time [22], compassion [23], good communication and adhering to the instructions of the medical staff [24], and trust in the medical staff as proven by Yuxian et al. in their research using 12 hospitals in China [25] might reduce violence against the medical staff.

In a certain way, this study hopes to reinforce what other writers have written in previous literatures on the causes of violence against medical staff. However, improving all of these parameters may not abolish violence completely, since patients with psychiatric or social background caused 10 of 19 violence cases during the COVID-19 outbreak and 31 of 51 cases in the same period in 2019. This population may not be aggressive to the medical staff; however, their character could affect their behavior.

## Conclusions

The presence of an external threat increases group cohesion if two conditions are met: if agreement among group members that the aggregate is a group and its preservation is worthwhile and if the perceived threat is against the group as a whole [26]. In line with others research, in a certain way, as thought by the authors, the COVID-19 outbreak may be an artificial model that united citizens and staff that positively influenced many conditions, mainly those related to compassionate staff and less work overload, which may lead to violence in some ordinary days. This model may give rise to some questions about both parties’ behavior and give some ideas to health policy decision-makers.

### Limitations of the study 

This study was conducted in a 1017 beds tertiary hospital. However, it was a small-scale study. Further studies from other hospitals in Israel or other countries are needed to confirm the hypothesis of this study. Besides, except for verbal and physical violence, there were other cases that defined as disruption of order. There are no exact details of what these disruptions include. However, it is known that these are not cases of violence against hospital staff and it was assumed that most of them were not influenced by the COVID-19 outbreak. Further investigation may be needed.

## Data Availability

The data of this study can be shared with others as needed on referral to our hospital security department.

## Abbreviations

ED: Emergency Department
MOH: Ministry of Health
BI: Business Intelligence
LOS: Length of Stay
IRB: Institutional review board
